# The SMOC1 proteomics network M42 controls multiple signaling modalities, brain homeostasis and toxicity in fly Alzheimer’s disease models

**DOI:** 10.1002/alz.089554

**Published:** 2025-01-03

**Authors:** David Li‐Kroeger, Ismael Al‐Ramahi, Nathaniel Smith, Aditi Marella, Bismark K Amoh, Saathwik Saladi, Juan Botas, Nicholas T Seyfried, Allan I. Levey, Joshua M Shulman

**Affiliations:** ^1^ Baylor College of Medicine ‐ NRI, Houston, TX USA; ^2^ Baylor College of Medicine, Houston, TX USA; ^3^ Jan and Dan Duncan Neurological Research Institute, Texas Children’s Hospital, Houston, TX USA; ^4^ Brandeis Universtiy, Waltham, MA USA; ^5^ Des Moines University, Des Moines, IA USA; ^6^ University of California, Los Angeles, CA USA; ^7^ Rice University, houston, TX USA; ^8^ Emory University School of Medicine, Atlanta, GA USA

## Abstract

**Background:**

Alzheimer’s disease (AD) has a complex etiology where insults in multiple pathways conspire to disrupt neuronal function, yet molecular changes underlying AD remain poorly understood. Previously, we performed mass‐spectrometry on post‐mortem human brain tissue to identify >40 protein co‐expression modules correlated to AD pathological and clinical traits. Module 42 has the strongest correlation to AD pathology and consists of 32 proteins including SMOC1, a predicted driver of network behavior and potential biomarker for AD. SMOC1 is a matrisomal protein with conserved roles in modulating TGF‐Beta and wnt signaling during development, yet remains unstudied in the brain.

**Methods:**

We evaluate M42 using a high‐throughput robotic screening platform and video assisted software, enabling quantitative assessments of neurological function to identify proteins that modify Aβ‐ or tau‐induced neurodegeneration. We then focus on dSMOC1 using *Drosophila* genetics and cell biological approaches to determine its role in the brain. Finally, we use Mass Spectrometry to identify protein changes in the brains of dSMOC1^‐/‐^ flies.

**Results:**

Our screening assay identified 20 genes from M42 that modify tau toxicity and 5 for Aβ, including dSMOC1 and components of Wnt and TGF‐β signaling pathways. We find dSMOC1^‐/‐^ null flies suffer severely decreased survival and climbing defects upon aging. In the brain, *dSMOC1* expression occurs primarily in glial cells while protein localizes around neuronal cell bodies, consistent with its role as a matrisomal protein. We show alteration of glypican levels in dSMOC1^‐/‐^ brains. Glypicans modulate Wnt and TGF‐β signaling further supporting a role connecting M42 in AD biology. Finally, we used mass spectrometry to determine protein changes in brains of dSMOC1^‐/‐^ flies and found perturbations in ECM/receptor interactions, proteostasis, KREBs/TCA cycle, and oxidative phosphorylation. Additional data suggests an age dependent shift from oxidative phosphorylation to glycolysis may compensate for reduced ATP levels in dSMOC1^‐/‐^ flies.

**Conclusion:**

The M42 protein module contains multiple proteins with links to AD. Multiple M42 proteins interact with specific AD triggers including SMOC1, wnt and TGF‐β signaling components. The genetics, proteomics, and cell biological data combine to support a mechanistic hypothesis where changes in SMOC1 levels disrupt critical signaling pathways leading to disruptions in metabolism affecting neuronal function.

